# Supplementary material for: The effect of intrapartum prolonged oxygen exposure on fetal metabolic status: secondary analysis from a randomized controlled trial
Source: Front Endocrinol (Lausanne). 2023 Jun 27;14:1204956. doi: 10.3389/fendo.2023.1204956 (PMC10335765; doi:10.3389/fendo.2023.1204956)
Supplement: Supplementary file 3 [file DataSheet_3.zip › Result-X101SC21092977-Z01-J001-B1-42/2.MetAnnotation/Lipidmaps/meta_neg.Lipidmaps.Anno.pdf]

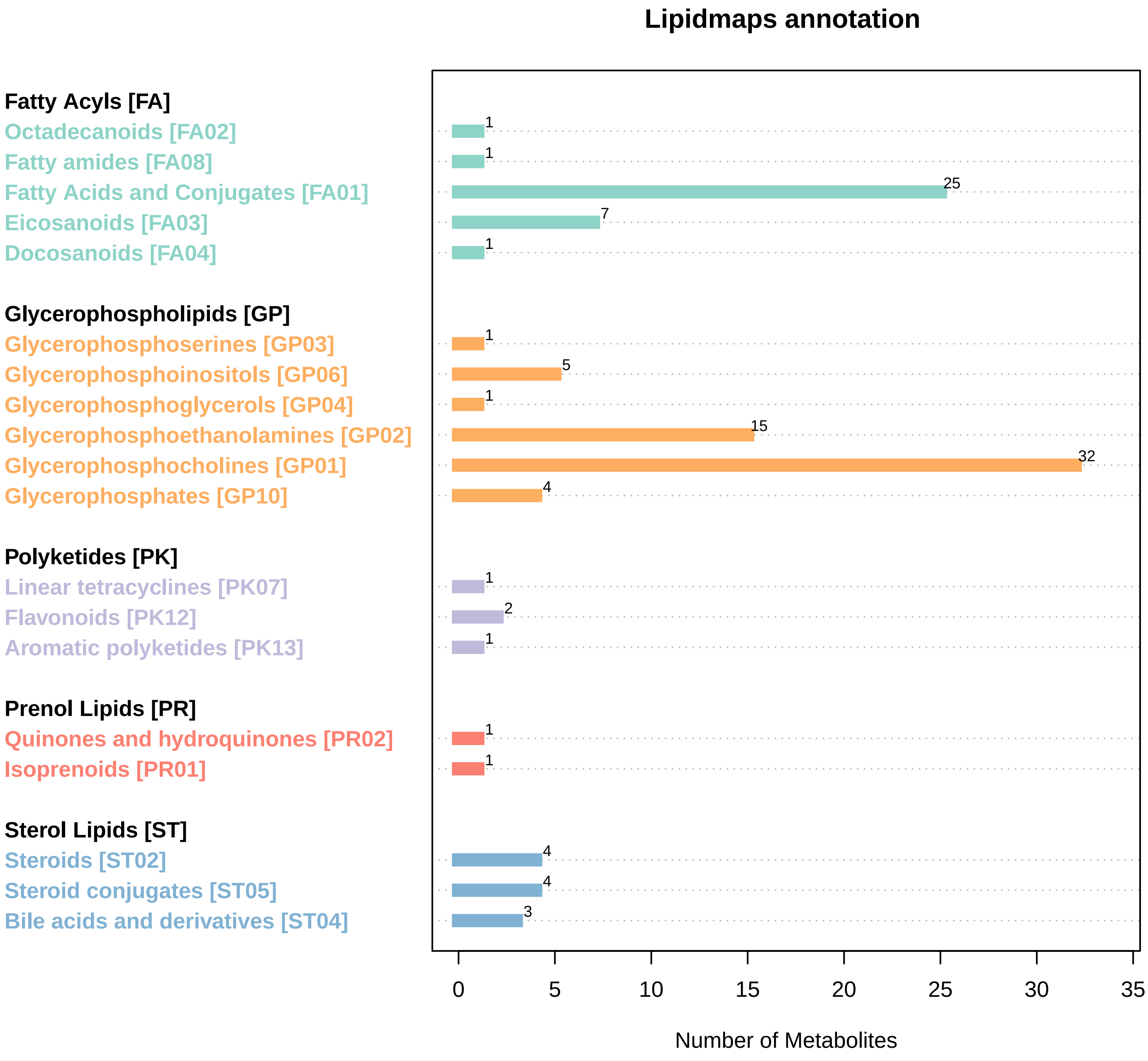

Lipidmaps annotation
Fatty Acyls [FA]
Octadecanoids [FA02]
Fatty amides [FA08]
Fatty Acids and Conjugates [FA01]
Eicosanoids [FA03]
Docosanoids [FA04]
Glycerophospholipids [GP]
Glycerophosphoserines [GP03]
Glycerophosphoinositols [GP06]
Glycerophosphoglycerols [GP04]
Glycerophosphoethanolamines [GP02]
Glycerophosphocholines [GP01]
Glycerophosphates [GP10]
Polyketides [PK]
Linear tetracyclines [PK07]
Flavonoids [PK12]
Aromatic polyketides [PK13]
Prenol Lipids [PR]
Quinones and hydroquinones [PR02]
Isoprenoids [PR01]
Sterol Lipids [ST]
Steroids [ST02]
Steroid conjugates [ST05]
Bile acids and derivatives [ST04]
1
1
25
7
1
1
5
1
15
32
4
1
2
1
1
1
4
4
3
0
5
10
15
20
25
30
35
Number of Metabolites
